# Endothelial NADPH oxidase 4 protects against angiotensin II‐induced cardiac fibrosis and inflammation

**DOI:** 10.1002/ehf2.13228

**Published:** 2021-01-29

**Authors:** Minshu Wang, Colin E. Murdoch, Alison C. Brewer, Aleksandar Ivetic, Paul Evans, Ajay M. Shah, Min Zhang

**Affiliations:** ^1^ School of Cardiovascular Medicine and Sciences James Black Centre, King's College London British Heart Foundation Centre of Excellence 125 Coldharbour Lane London SE5 9NU UK; ^2^ Department of Ophthalmology Peking University Third Hospital Beijing China; ^3^ Infection, Immunity and Cardiovascular Disease University of Sheffield Medical School Sheffield UK

**Keywords:** Myocardial fibrosis, Nox4, Endothelial dysfunction, Inflammation, Angiotensin II

## Abstract

**Aims:**

Endothelial activation and inflammatory cell infiltration have important roles in the development of cardiac fibrosis induced by renin–angiotensin system activation. NADPH oxidases (Nox proteins) are expressed in endothelial cells (ECs) and alter their function. Previous studies indicated that Nox2 in ECs contributes to angiotensin II (AngII)‐induced cardiac fibrosis. However, the effects of EC Nox4 on cardiac fibrosis are unknown.

**Methods and results:**

Transgenic (TG) mice overexpressing endothelial‐restricted Nox4 were studied alongside wild‐type (WT) littermates as controls. At baseline, Nox4 TG mice had significantly enlarged hearts compared with WT, with elongated cardiomyocytes (increased by 18.5%, *P* < 0.01) and eccentric hypertrophy but well‐preserved cardiac function by echocardiography and *in vivo* pressure–volume analysis. Animals were subjected to a chronic AngII infusion (AngII, 1.1 mg/kg/day) for 14 days. Whereas WT/AngII developed a 2.1‐fold increase in interstitial cardiac fibrosis as compared with WT/saline controls (*P* < 0.01), TG/AngII mice developed significant less fibrosis (1.4‐fold increase, *P* > 0.05), but there were no differences in cardiac hypertrophy or contractile function between the two groups. TG hearts displayed significantly decreased inflammatory cell infiltration with reduced levels of vascular cell adhesion molecule 1 in both the vasculature and myocardium compared with WT after AngII treatment. TG microvascular ECs stimulated with AngII *in vitro* supported significantly less leukocyte adhesion than WT ECs.

**Conclusions:**

A chronic increase in endothelial Nox4 stimulates physiological cardiac hypertrophy and protects against AngII‐induced cardiac fibrosis by inhibiting EC activation and the recruitment of inflammatory cells.

## Highlights

- Mice with endothelium‐specific overexpression of Nox4 (EndoNox4 TG) exhibit eccentric hypertrophy with well‐preserved cardiac function at baseline.
- EndoNox4 TG mice develop significantly less interstitial cardiac fibrosis in response to chronic pressure AngII stimulation, independent of cardiac hypertrophy.
- Overexpression of Nox4 in endothelial cells reduces AngII‐induced endothelial activation.
- An increase in endothelial Nox4 inhibits AngII‐induced recruitment of inflammatory cells in the heart.

## Introduction

Myocardial fibrosis involving the excessive deposition of extracellular matrix and accumulation of abnormally cross‐linked collagen alters both the geometry and mechanical properties of the heart over time and constitutes an important component of adverse cardiac remodelling.^1^ The extent of myocardial fibrosis is associated with higher mortality in patients with heart failure. However, current treatment regimens are ineffective in reducing or reversing fibrosis,^2^ largely because the fundamental mechanisms underlying the fibrotic progression remain incompletely understood.

Activation of endothelial cells (ECs), in particular via increased expression of adhesion receptors such as vascular cell adhesion molecule 1 (VCAM‐1) that recruit circulating inflammatory cells and the localized secretion of cytokines,^3^ is involved in cardiac fibrosis. Increased activation of the renin–angiotensin–aldosterone system (RAAS) plays a central role in pro‐inflammatory and pro‐fibrotic actions and modulating endothelial function. Growing evidence indicates that these effects are strongly regulated through redox‐sensitive processes, particularly the activation of NADPH oxidases (Nox), which are major sources of reactive oxygen species (ROS) in cardiovascular system.^4^

Of the seven Nox isoforms (Nox1–5, Duox1–2) that have been identified so far, Nox2 and Nox4 are the most abundantly expressed in the heart. Previous studies found that Nox2 is involved in the pathophysiology of angiotensin II (AngII)‐dependent endothelial dysfunction and the development of cardiac fibrosis. Interstitial fibrosis induced by activation of the renin–angiotensin system was inhibited in Nox2 knockout mice or in cardiomyocyte‐specific Rac1 knockout mice (which have deficient Nox2 activation).^5^, ^6^, ^7^ The finding that deletion of Nox2 may attenuate fibrosis without altering the extent of cardiomyocyte hypertrophy suggested that the effects of Nox2 may involve a cell type extrinsic to myocytes.^6^, ^8^ Indeed, our studies revealed that endothelial Nox2 contributes to AngII‐induced cardiac fibrosis through pro‐inflammatory effect via induction of VCAM‐1 and increased endothelial–mesenchymal transition.^8^

In contrast to Nox2, Nox4 is constitutively active and is regulated mainly by its level of abundance.^9^ Using global Nox4 knockout mice and cardiomyocyte‐specific transgenic (TG) mouse models, our previous studies showed that Nox4 is protective against cardiac hypertrophy, contractile dysfunction, and fibrosis in response to pressure overload or myocardial ischaemia. Different cardiomyocyte‐based adaptive mechanisms may be involved including a paracrine preservation of myocardial capillary density,^10^ Nrf2‐dependent modulation of redox state,^11^ and enhancement of the integrated stress response.^12^ Nox4 is highly expressed in the endothelium,^13^ but the role of endothelial Nox4 in cardiac fibrosis and remodelling has not been investigated.

In this study, we employed a previously generated TG mouse model with endothelial‐targeted overexpression of Nox4 (EndoNox4 TG)^14^ to evaluate the effects of endothelial Nox4 on AngII‐induced cardiac fibrosis.

## Material and methods

### Animal studies

All procedures were performed in accordance with the Guidance on the Operation of the Animals (Scientific Procedures) Act, 1986 (UK Home Office). Endothelium‐targeted Nox4 overexpression (EndoNox4 TG) using an established tie2 promoter construct was described previously.^14^ Mice were backcrossed onto a C57BL/6 background for >10 generations. We studied male mice aged 8–16 weeks and matched wild‐type (WT) littermates.

Angiotensin II (1.1 mg/kg/day) or saline vehicle was infused via subcutaneously implanted osmotic minipumps (Model 1002, Alzet, Cupertino, CA) for 14 days. Echocardiography was performed under 2% isoflurane anaesthesia with heart rates maintained >400 b.p.m. using a Vevo 2100 machine with a 30 MHz linear array transducer (Visualsonics, Toronto, CA).^15^ Left ventricular (LV) pressure–volume (PV) relations were measured with a 1.4F microconductance catheter system (SPR‐839, Millar Instruments, Houston, TX) introduced retrogradely into the LV via the right carotid artery under 2% isoflurane anaesthesia.^8^

### Assessment of isolated cardiomyocyte size

Fresh ventricular myocytes were isolated from mouse hearts as described.^15^ Isolated cardiomyocytes suspended in medium were analysed in a Coulter counter‐analyser system (Multisizer 3 Coulter Counter, Beckman Coulter, Inc., USA) to measure the cell volume. Cardiomyocytes were also plated on slides, and images were obtained under light microscopy (Zeiss Axioscope, Germany) to evaluate cell length.^16^

### Immunoblotting, histology, and immunohistochemistry

Proteins were extracted and Western blots performed using standard procedures. Cardiomyocyte area and cardiac fibrosis were quantified in 6 μm paraffin LV sections stained with wheat‐germ agglutinin (WGA) and Picrosirius Red, respectively.^10^ Collagen content was calculated as % of total LV area. Capillaries were stained with isolectin B4 (Vector B‐1205), and capillary density was quantified as the number of capillaries per square millimetre.^10^ Immunostaining was performed using primary antibodies and HRP‐labelled secondary antibodies, with visualization by diaminobenzidine (DAKO kit SK4100, Vector Laboratories). CD45‐positive, CD3‐positive, or Mac3‐positive cells were counted using semi‐automated software (AxioVision v4.6, Carl Zeiss, Germany) and expressed as average counts over 10 fields. The following antibodies were used: anti‐VCAM‐1 (AF643, R&D Systems), anti‐β‐actin (ab8227, Abcam), anti‐CD45 (550539, BD Biosciences), anti‐Mac3 (550292, BD Biosciences), and anti‐CD3 (ab5690, Abcam).

### Real‐time PCR

mRNA expression levels were quantified by real‐time RT–PCR using SYBR Green on an Applied Biosystems PRISM 7700 machine. Data were analysed using the comparative Ct method and normalized by β‐actin levels. Primer sequences were (forward, reverse): *Anf*, atrial natriuretic factor: ATTGGAGCCCACAGTGGACTA, CCTTTTCCTCCTTGGCTGTTATC; *P1np*, type I procollagen: CCTCAGGGTATTGCTGGACAAC, TTGATCCAGAAGGACCTTGTTTG; *P3np*, type III procollagen: AGGAGCCAGTGGCCATAATG, TGACCATCTGATCCAGGGTTTC; *Fn*, fibronectin: CCGGTGGCTGTCAGTCAGA, CCGTTCCCACTGCTGATTTATC; *Tnf‐α*, tumour necrosis factor α: GTTCTATGGCCCAGACCCTCA, TCCACTTGGTGGTTTGCTACG; *Il‐6*, interleukin‐6: GAAAAGAGTTGTGCAATGGCAAT, TTGGTAGCATCCATCATTTCTTTG.

### *En face* immunofluorescence staining

The expression levels of VCAM‐1 were assessed in ECs at regions of the lesser curvature [low shear (LS) site], greater curvature [high shear (HS) site], and descending mouse aortae by *en face* staining as described previously.^17^ Briefly, mice were treated with AngII (1.1 mg/kg/day) or saline for 48 h before killing by CO_2_ inhalation. Mice were slowly perfused with cold phosphate‐buffered saline and then perfusion‐fixed with 2% formalin before harvesting. Aortae were cut longitudinally along the greater curvature to reveal the endothelial surface and tested by immunostaining using primary antibody against VCAM‐1 or isotype IgG as a negative control and Alexa Fluor 568‐conjugated secondary antibodies (red). ECs were identified by co‐staining using anti‐CD31 antibody (CBL1337, Chemicon) conjugated to the fluorophore FITC, and nuclei were co‐stained using Draq5 (BioStatus). Fluorescence images were taken using confocal laser scanning microscopy (Zeiss LSM 510 META, Germany). The expression of VCAM‐1 was assessed by quantification of fluorescence intensity of four random images of each site using Velocity software in a blinded manner.

### Leukocyte adhesion flow assay

Coronary microvascular endothelial cells (CMECs) were isolated from hearts of 6‐ to 8‐week‐old mice and used at Passages 2–3.^8^ Interaction between CMECs and leukocytes was measured by flow assay as reported previously.^8^ Briefly, a parallel plate flow chamber (GlycoTech, Maryland) was assembled with confluent CMEC monolayers, which had been previously incubated with or without AngII (100 nmol/L) for 4 h. Bone marrow cells were isolated from WT mice and labelled with CellTracker dye (Invitrogen) prior to perfusion over stimulated CMEC monolayers at a cell density of 1 × 10^6^ cells/mL and a shear stress of 2.5 dyn/cm^2^ for 30 min. The first 10 min of perfusion was with bone marrow cell suspension, and the subsequent 20 min was without cells. Non‐bound leukocytes were eventually cleared with continued perfusion of media lacking cells. The remaining adherent cells were visualized using an inverted time‐lapse fluorescence microscope (Olympus IX81). The number of recruited leukocytes was counted in 8–10 fields of view per flow assay performed on four separate occasions.

### Statistics

Data are presented as mean ± SEM. Comparisons between TG and WT were made by unpaired Student's *t*‐test for two groups. Two‐way ANOVA was used to compare treatment responses between TG and WT followed by Bonferroni post‐tests. Analyses were performed on GraphPad Prism (8.0.0 for Windows, San Diego, CA). *P* < 0.05 was considered statistically significant.

## Results

### EndoNox4 transgenic mice exhibit eccentric cardiac hypertrophy at baseline

**Figure 1:**
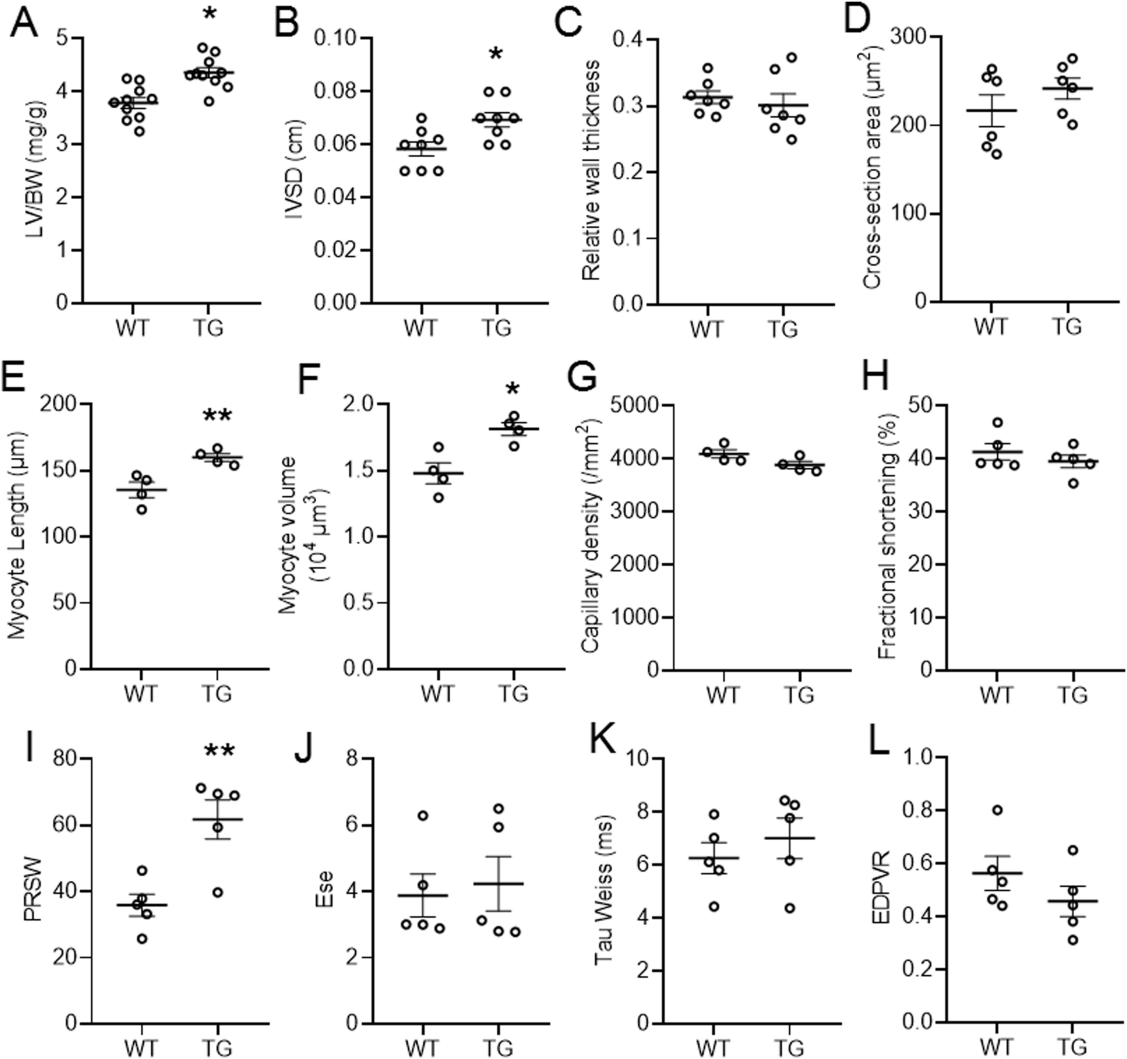
EndoNox4 TG mice exhibit physiological cardiac hypertrophy. (A) Left ventricle/body weight ratios (LV/BW). *n* = 10 mice/group. (B–C) Cardiac hypertrophy evaluated by echocardiography. IVSD, interventricular septum thickness at end‐diastole. Relative wall thickness: (IVSD + post wall diameter)/LVEDD as indicator of concentric hypertrophy. *n* = 8 mice/group. (D) Quantification of transverse heart cross‐sectional area for cardiomyocyte width determination. *n* = 6 hearts/group. (E) Quantification of isolated cardiomyocyte length. *n* = 4 hearts/group. (F) Mean data of cell volumes assessed on a Coulter counter. *n* = 4 hearts/group. (G) Mean data for capillary density. *n* = 4 hearts/group. (H–J) Cardiac systolic function measured by echo and PV loop analysis. *n* = 5 mice/group. Ees, end‐systolic elastance; PRSW, preload recruitable stroke work. (K–L) Cardiac diastolic function evaluated by PV loop analysis. EDPVR, end‐diastolic pressure–volume relationship. *n* = 5 mice/group. *P < 0.05, **P < 0.01, compared with WT mice, unpaired Student's *t*‐test. All data are mean ± SEM.

EndoNox4 TG mice showed no obvious abnormalities at baseline and bred normally, as described previously.^14^ We first examined the effect of endothelial‐specific Nox4 overexpression on basal cardiac morphology and function. EndoNox4 TG mice exhibited enlarged hearts in terms of higher LV weight/body weight ratio (*Figure* *1* *A*). Echocardiography revealed that EndoNox4 TG mouse hearts had significantly increased intraventricular septal thickness in diastole (IVSD) compared with WT littermates (*Figure* *1* *B*). However, LV relative wall thickness (RWT), defined as the ratio of IVSD plus posterior LV wall thickness to the LV internal diameter at end‐diastole (LVEDD), was similar in TG and WT hearts^18^ (*Figure* *1* *C*). This finding suggested an eccentric pattern of hypertrophy in TG hearts, a phenotype similar to physiological cardiac hypertrophy. The cardiomyocyte cross‐sectional area in heart sections indicated that the myocyte width was not significantly different between TG and WT (*Figure* *1* *D*). The length of isolated cardiomyocytes, however, was significantly increased by 18% in TG mice (*Figure* *1* *E*). Consistent with these data, the average volume of cardiomyocytes was significantly greater in TG mice compared with WT (*Figure* *1* *F*). There was no difference in myocardial capillary density between the genotypes (*Figure* *1* *G*).

We next evaluated the cardiac functional consequences of the eccentric hypertrophy. Echocardiographic fractional shortening (FS) was similar in EndoNox4 TG and WT hearts (*Figure* *1* *H*). Cardiac contractility was further assessed by *in vivo* pressure–volume analysis. TG mice displayed slightly increased indices of LV systolic function as evidenced by significantly higher preload recruitable stroke work (PRSW) (*Figure* *1* *I*) but with an unaltered end‐systolic elastance (Ees) (*Figure* *1* *J*). Both the isovolumic relaxation time constant, *τ*, and the LV end‐diastolic pressure volume relationship (EDPVR) were similar between EndoNox4 TG and WT mice (*Figure* *1* *K* and *1* *L*).

Taken together, these results indicate that EndoNox4 TG mice exhibit baseline eccentric hypertrophy with a physiological pattern of slightly enhanced LV systolic function and no impairment of diastolic function.

### EndoNox4 transgenic and wild‐type mice develop similar extent of cardiac hypertrophy in response to angiotensin II

**Figure 2:**
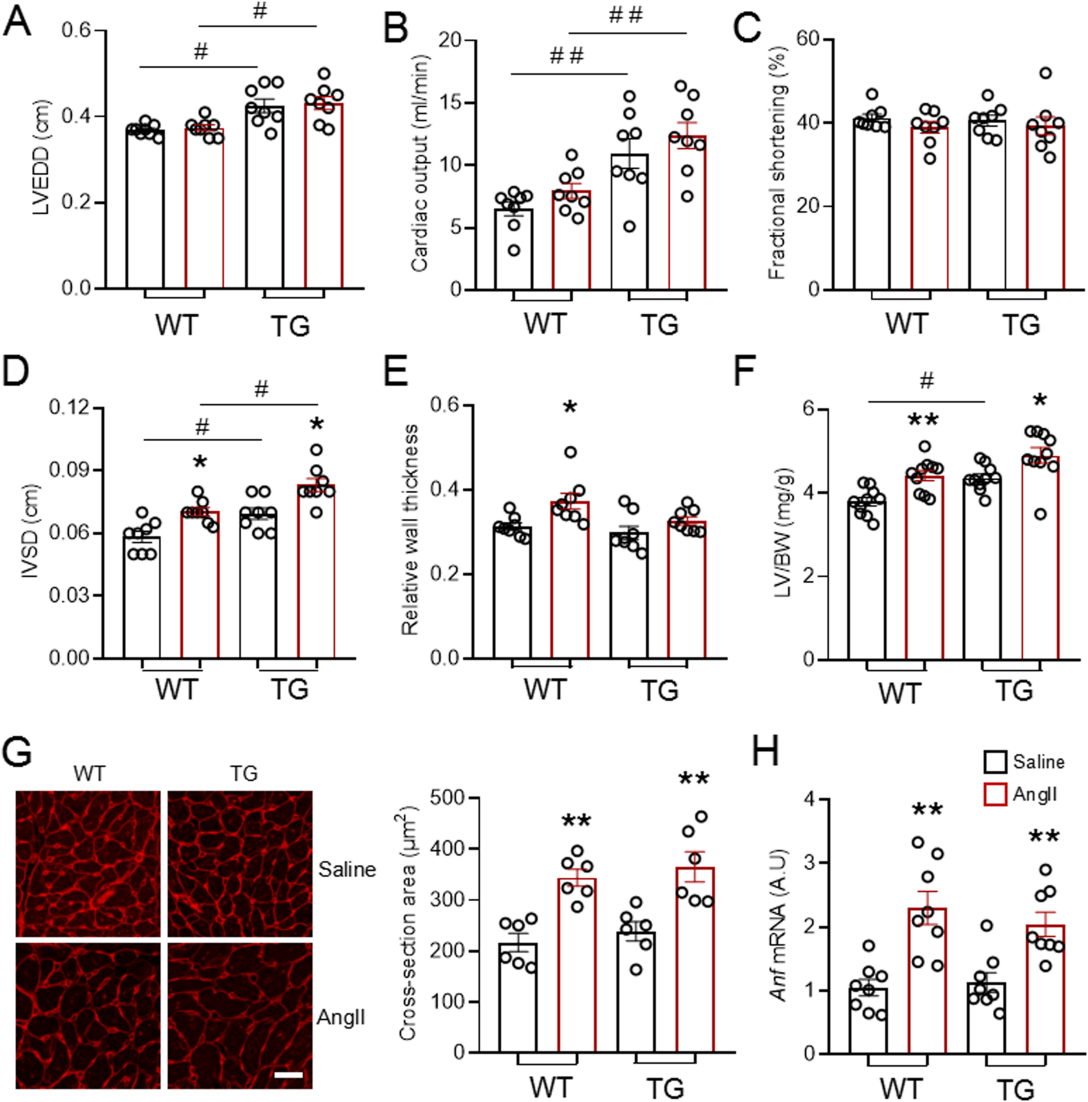
EndoNox4 TG mice develop similar extent of cardiac hypertrophy as WT in response to AngII stimulation. (A–C) Cardiac function assessed by echocardiography. LVEDD, left ventricular end‐diastolic dimension. *n* = 8 mice/group. (D–E) Echocardiographic measurement of interventricular septum thickness at end‐diastole (IVSD) and relative wall thickness. *n* = 8 mice/group. (F) LV weight and body weight ratios. *n* = 10 mice/group. (G) Representative transverse heart sections for cardiomyocyte area determination stained with WGA. Scale bars: 20 μm. Mean data shown on the right. *n* = 6 hearts/group. (H) mRNA level of hypertrophic marker atrial natriuretic factor (*Anf*). *n* = 8 hearts/group. *P < 0.05, **P < 0.01 compared with respective saline groups; ^#^ *P* < 0.05, ^##^ *P* < 0.01 compared with respective WT mice; two‐way ANOVA with post‐Bonferroni tests. All data are mean ± SEM.

EndoNox4 TG and WT littermates were subjected to chronic AngII infusion for 2 weeks (1.1 mg/kg/day). Echocardiographic evaluation showed that EndoNox4 TG had a larger LV end‐diastolic dimension (LVEDD) and cardiac output than WT mice both before and after AngII infusion, with no effect of AngII itself (*Figure* *2* *A* and *2* *B*). This is consistent with the baseline phenotype of eccentric cardiac hypertrophy in TG mice. Similarly, AngII infusion did not significantly change the LV end‐systolic dimension (LVESD) in either group (data not shown), and overall cardiac function as assessed by FS was also unchanged (*Figure* *2* *C*). Cardiac hypertrophy as assessed by the IVSD was induced by AngII in both WT and TG mice, but the magnitude of increase was similar between genotypes (*Figure* *2* *D*). However, the RWT only increased in WT mice treated with AngII, consistent with a pattern of concentric hypertrophy, but remained unaltered in TG/AngII mice (*Figure* *2* *E*). The results for cardiac hypertrophy were confirmed by the measurement of LV weight/body weight ratio and showed a similar increase in AngII‐treated WT and TG mice (*Figure* *2* *F*). Similarly, at a cellular level, cardiomyocyte hypertrophy as assessed by cross‐sectional area increased to an equivalent extent in WT and TG hearts (*Figure* *2* *G*). We also quantified changes in the myocardial mRNA levels of *Anf*, which typically increases with pathological hypertrophy. There was no difference in mRNA expression of *Anf* between EndoNox4 TG and WT at baseline, while after AngII infusion the levels increased to a similar extent in both groups (*Figure* *2* *H*). These data indicate that while an increase in endothelial Nox4 induces baseline eccentric hypertrophy, the hypertrophic response to chronic AngII stimulation is similar between genotypes.

### Endothelial Nox4 protects against angiotensin II‐induced myocardial fibrosis

**Figure 3:**
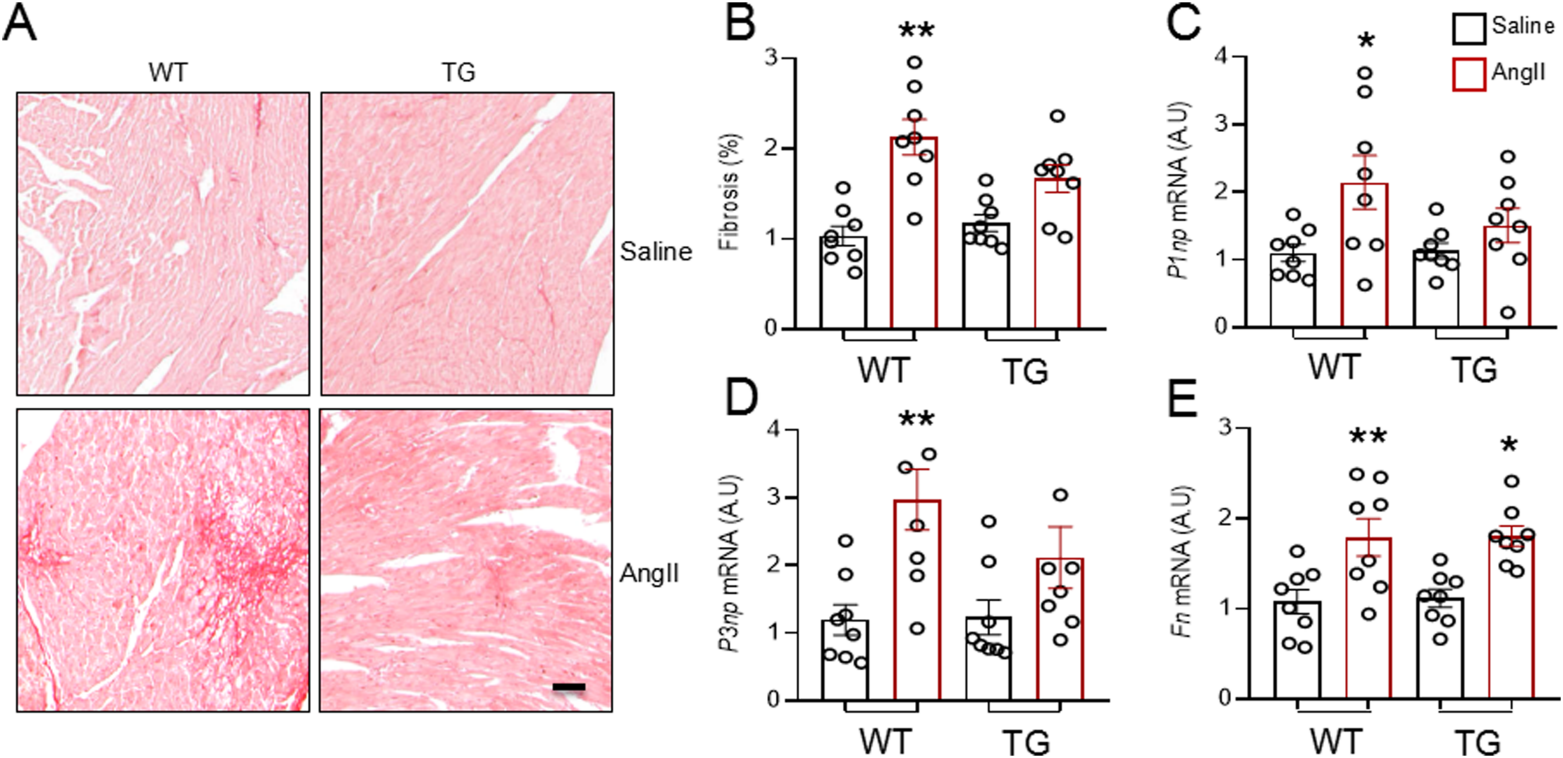
Overexpression of endothelial Nox4 attenuates AngII‐induced myocardial fibrosis. (A) Representative images of myocardial interstitial fibrosis by Picrosirius Red staining. Scale bar: 100 μm. (B) Quantification of myocardial fibrosis. *n* = 8 hearts/group. (C–E) Quantification of mRNA levels of fibrotic markers. *n* = 8 hearts/group. *P < 0.05, **P < 0.01 compared with respective saline groups; two‐way ANOVA with post‐Bonferroni tests. All data are mean ± SEM.

Myocardial fibrosis was assessed in LV sections stained with Picrosirius Red. The level of fibrosis was very low in both saline‐treated WT and TG mouse hearts, suggesting that an increased level of Nox4 in ECs does not have significant effects on the extracellular matrix at baseline (*Figure* *3* *A* and *3* *B*). Two‐week AngII infusion significantly enhanced myocardial fibrosis by 2.1‐fold in WT hearts. However, this increase was markedly blunted (to around 1.4‐fold) in TG mouse hearts after AngII treatment (*Figure* *3* *A* and *3* *B*). At a molecular level, AngII markedly increased the gene expression of the pro‐fibrotic genes *P1np* and *P3np* in WT mouse hearts, but this was significantly blunted in TG/AngII mice (*Figure* *3* *C* and *3* *D*). The mRNA levels of fibronectin increased to a similar extent in both WT and TG hearts (*Figure* *3* *E*). These data suggest that endothelial Nox4 is protective against AngII‐induced myocardial fibrosis.

### Endothelial Nox4 reduces angiotensin II‐induced inflammatory cell infiltration in the heart

**Figure 4:**
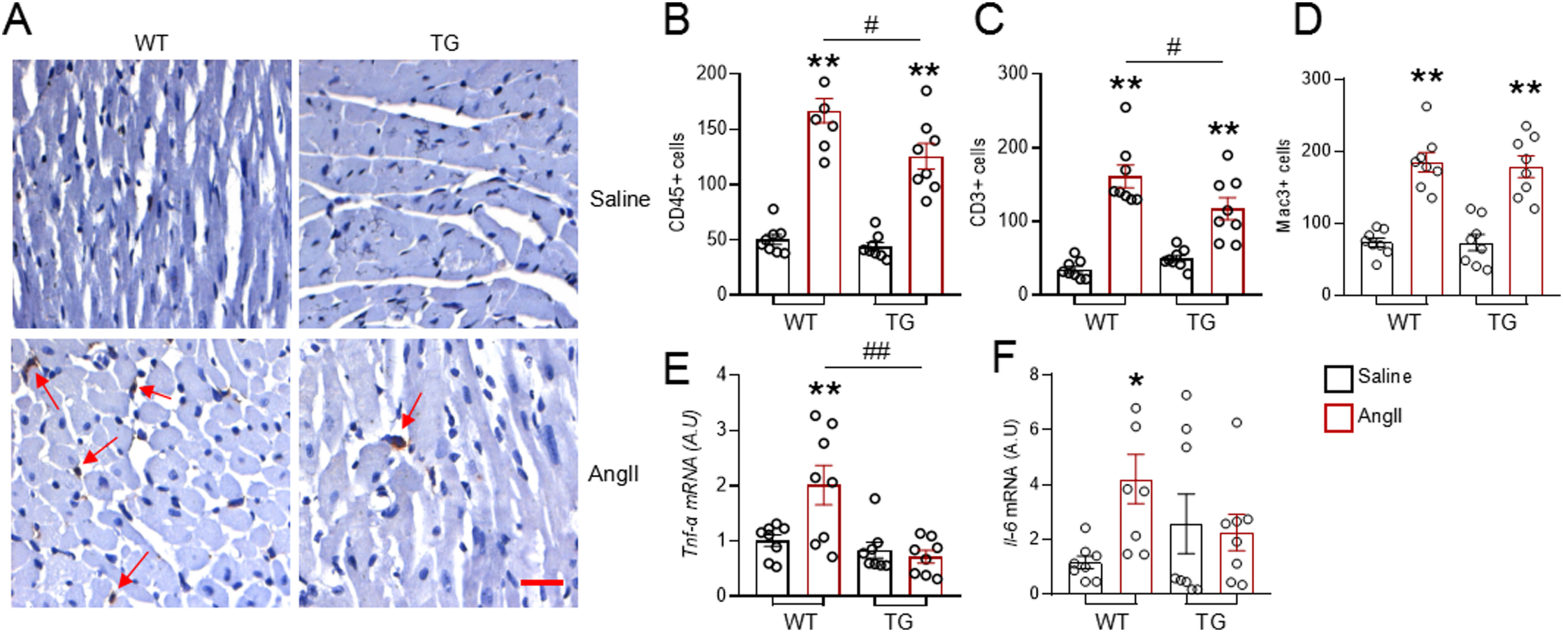
Overexpression of endothelial Nox4 alleviates AngII‐induced inflammatory cell infiltration in the heart. (A) Typical immunostaining images of CD45 inflammatory cells. Scale bar: 25 μm. Arrows indicate CD45‐positive cells shown as brown colour and counter‐stained with Harris solution. (B–D) Quantifications of CD45‐positive, CD3‐positive, and Mac3‐positive cells. *n* = 8 hearts/group. (E–F) mRNA levels of pro‐inflammation genes tumour necrosis factor α (*Tnf‐α*) and interleukin 6 (*Il‐6*) in the heart. *n* = 8 hearts/group. *P < 0.05, **P < 0.01 compared with respective saline groups. ^#^ *P* < 0.05, ^##^ *P* < 0.01 compared with WT/AngII; two‐way ANOVA with post‐Bonferroni tests. All data are mean ± SEM.

To investigate mechanisms underlying the attenuated fibrosis in EndoNox4 TG hearts, we assessed myocardial inflammatory cell infiltration, which is known to contribute to AngII‐induced cardiac fibrosis.^8^ As expected, AngII treatment significantly increased CD45 (pan‐leukocyte marker)‐positive and CD3 (T‐cell marker)‐positive cells in WT hearts (*Figure* *4* *A*–*4* *C*). TG hearts, however, had significantly less CD45^+^ inflammatory cells and CD3^+^ cells after AngII infusion than WT hearts (*Figure* *4* *A*–*4* *C*). The numbers of Mac3 (macrophage marker)‐positive cells were elevated to a similar extent in both WT and TG hearts after AngII infusion (*Figure* *4* *D*). AngII treatment also significantly increased the mRNA levels of several pro‐inflammatory cytokines including tumour necrosis factor α (*Tnf‐α*) (*Figure* *4* *E*) and interleukin 6 (*Il‐6*) (*Figure* *4* *F*) in WT hearts. However, these increases were virtually abolished in TG mice infused with AngII (*Figure* *4* *E* and *4* *F*).

### Nox4 attenuates endothelial activation by inhibition of vascular cell adhesion molecule 1 expression

**Figure 5:**
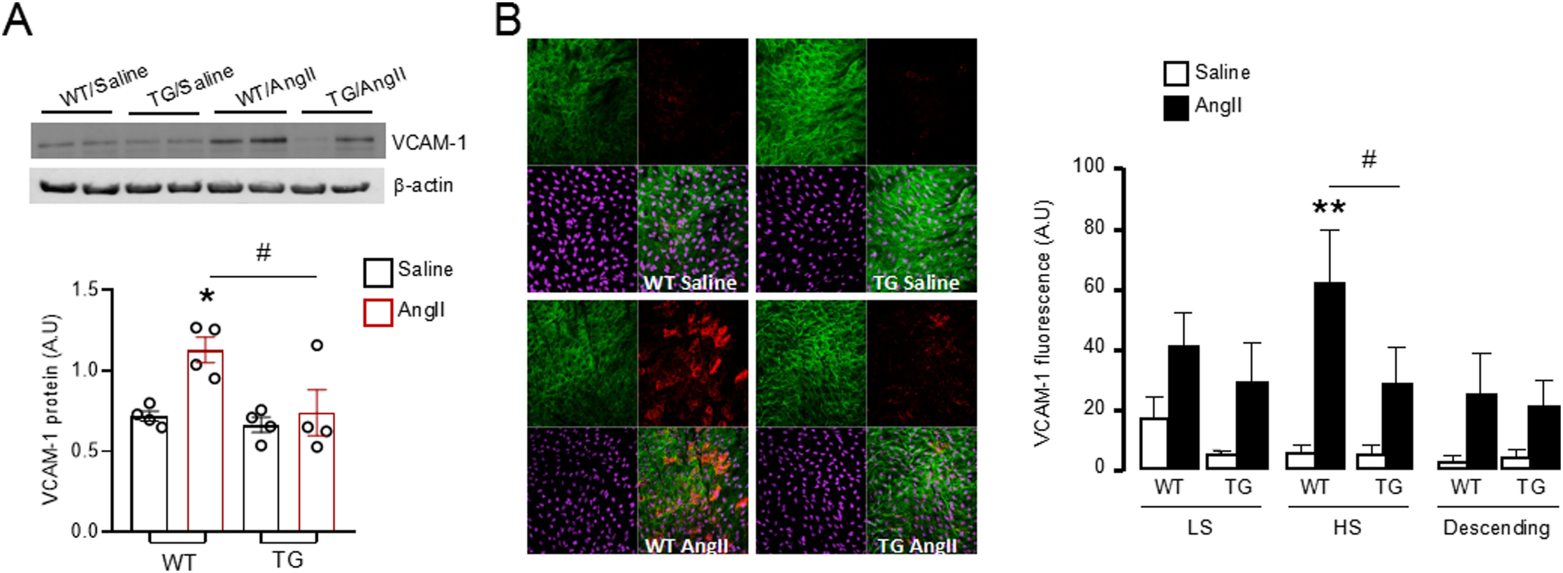
Forced expression of Nox4 in endothelial cells decreases VCAM‐1 expression by AngII stimulation. (A) Protein levels of VCAM‐1 in the heart by Western blot. Mean data shown at the below. *n* = 4 hearts/group. (B) Representative *en face* staining of VCAM‐1 in aortic greater curvature site [high shear (HS) region]. Green: CD31 staining for endothelial cells. Red: VCAM‐1 staining. Purple: Draq5 staining for nuclei. Mean data of fluorescence density of VCAM‐1 shown at the right. LS, low stress region. *n* = 4 vessels/group. *P < 0.05, **P < 0.01 compared with WT/saline. ^#^ *P* < 0.05 compared with WT/AngII; two‐way ANOVA with post‐Bonferroni tests. All data are mean ± SEM.

To further investigate the interrelationship between endothelial Nox4 and inflammatory cells, we first quantified the protein levels of VCAM‐1, an important endothelial‐expressed cell adhesion molecule involved in the recruitment of leukocytes. While there was no difference between genotypes after saline infusion, the increase in VCAM‐1 levels after chronic AngII infusion was significantly lower in TG hearts compared with WT (*Figure* *5* *A*).

To assess the effects of *in vivo* AngII infusion specifically on the endothelial levels of VCAM‐1 at an early stage, we employed *en face* staining of the aorta after 2 days of treatment with AngII or saline. Three regions of aorta were evaluated: LS region, HS region of aortic arch, and descending aorta. The levels of basal VCAM‐1 were very low in all aortic regions with no difference between WT and TG mice in saline‐treated groups. AngII stimulated an increase in VCAM‐1 levels, especially in the HS region of the WT aortic arch (*Figure* *5* *B*). In mice with endothelial‐specific overexpression of Nox4, the AngII‐induced up‐regulation of VCAM‐1 was markedly attenuated in the HS region (*Figure* *5* *B*). Similar trends were observed in the LS region and descending aorta but did not reach significance (*Figure* *5* *B*).

**Figure 6:**
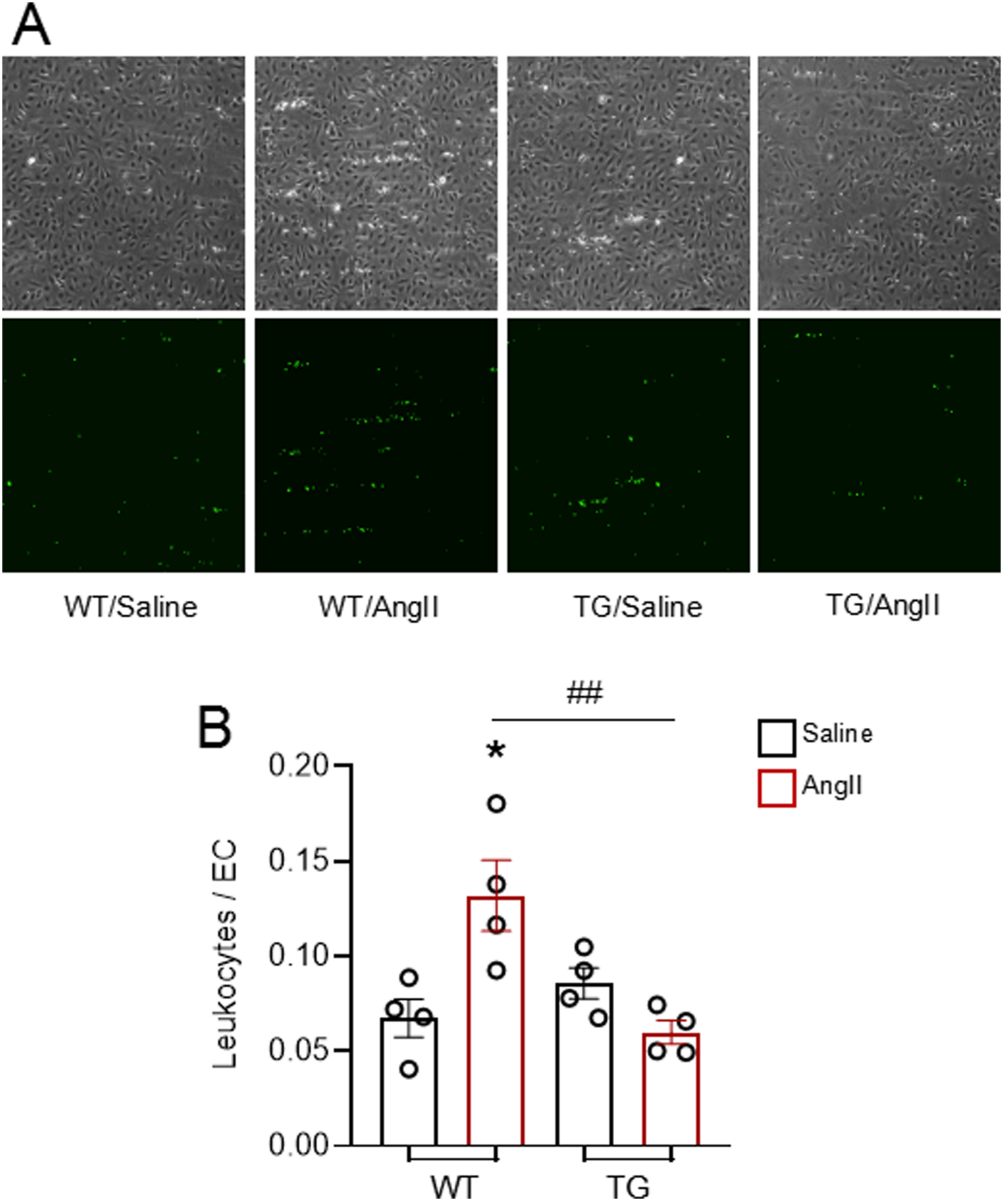
Endothelial Nox4 inhibits AngII‐induced endothelial activation. (A) Typical images of leukocytes binding to coronary microvascular endothelial cells (CMECs). Top panels: phase images; bottom panels: fluorescence images in which green spots indicate leukocytes adhered to underlying endothelial cells. (B) Quantification of bone marrow‐isolated leukocytes binding to CMECs after 30 min of continuous flow, expressed in the ratio of number of adhesive inflammatory cells towards number of endothelial cells in the same view. *n* = 4. *P < 0.05, compared with WT/saline. ^##^ *P* < 0.01 compared with WT/AngII; two‐way ANOVA with post‐Bonferroni tests. All data are mean ± SEM.

Finally, we assessed the functional interaction between inflammatory cells and Nox4‐overexpressing ECs in an *in vitro* adhesion assay under flow conditions. Leukocyte attachment to either WT or TG CMECs was low at baseline. After AngII treatment (100 nmol/L, 4 h), however, there was a significant increase in the number of leukocytes attached to WT CMECs (*Figure* *6*). This AngII‐induced increase was completely inhibited when CMECs from EndoNox4 TG mouse heart were used in the assay (*Figure* *6*). These findings suggest that endothelial Nox4 inhibits activation of EC and their interaction with leukocytes in the heart.

## Discussion

In this study, we found that an elevation of Nox4 levels in the endothelium significantly reduces the development of AngII‐induced myocardial fibrosis without affecting the extent of cardiac hypertrophy. Investigation of the underlying mechanisms revealed that endothelial Nox4 decreases VCAM‐1 expression in both the vasculature and myocardium and therefore reduces endothelial–leukocyte interaction. As a result, endothelial Nox4 protects against AngII‐induced myocardial inflammation and fibrosis. We also found that a constitutive increase in endothelial Nox4 is associated with modest eccentric cardiac hypertrophy with physiological characteristics, that is, preserved systolic and diastolic contractile function.

The heathy endothelium is considered to be a gatekeeper of cardiovascular homeostasis by maintaining anti‐inflammatory and anti‐thrombotic states.^19^ ECs can be activated by various pathologic stimuli, such as increased renin–angiotensin system activation or cytokine signalling. Activated ECs express pro‐inflammatory cell surface adhesion molecules such as VCAM‐1 and secrete pro‐inflammatory molecules such as TNF‐α and IL‐6, which act in concert to promote leukocyte adhesion and recruitment and perpetuate tissue inflammation.^20^ In the heart, sustained activation of ECs and associated pro‐inflammatory effects may enhance cardiac fibrosis and remodelling. Previous studies suggested that endothelial Nox‐derived ROS modulate VCAM‐1‐dependent lymphocyte migration.^21^ The importance of endothelial Nox2 in endothelial activation in response to chronic AngII stimulation was later demonstrated in a TG mouse model with EC‐targeted Nox2 overexpression.^8^, ^22^ EC Nox2 increased the number of inflammatory cells in the heart at least in part through an enhanced expression of VCAM‐1.^8^ In the current study, we find that mice with EC‐specific overexpression of Nox4 manifest opposite effects to those of Nox2, with a significant decrease in AngII‐stimulated VCAM‐1 expression in vessels and myocardium. This inhibition of VCAM‐1 expression was associated with reduced endothelial–leukocyte adhesion in an *in vitro* flow assay, as well as less inflammatory cell infiltration in the heart *in vivo* after AngII treatment. These results indicate that, in contrast to Nox2, an increase in Nox4 in ECs has a beneficial effect against AngII‐induced cardiac inflammation and fibrosis, at least in part by inhibiting endothelial activation. The mechanism whereby Nox4 down‐regulates VCAM‐1 was not addressed in the current study. However, it has previously been reported that endothelial activation is inhibited by the induction of the transcription factor Nrf2, which plays a critical role in the maintenance of endothelial homeostasis and resistance to AngII and cytokine‐induced injury.^23^, ^24^ Importantly, Nox4 is an essential modulator of Nrf2 activation in the vasculature^13^ and myocardium, both physiologically^25^ and in disease settings such as pressure overload.^11^

Reactive oxygen species play complex cell‐specific and context‐specific roles in pathophysiological processes such as cardiac remodelling. Considerable evidence implicates Nox2‐derived ROS in the development of cardiac remodelling, including interstitial fibrosis.^5^ While a reduction in AngII‐induced cardiac fibrosis was first identified in global Nox2 knockout mice, subsequent studies have shown that several different cell types play a role.^6^, ^26^ Evidence obtained with TG mice overexpressing Nox2 in ECs indicated a prominent contribution of endothelial Nox2 to AngII‐dependent cardiac fibrosis.^8^ A recent study showed that Nox2 in regulatory T cells may also act to enhance AngII‐induced cardiac fibrosis.^27^ Although a detrimental effect of Nox2 is supported by numerous studies, the role of Nox4 is unclear. Some studies showed that Nox4 in fibroblasts promoted cardiac fibrosis in response to RAAS activation^28^, ^29^ or TGF‐β stimulation.^30^ It was reported that a high level of cardiomyocyte‐specific Nox4 overexpression, with eight‐fold greater ROS production, exaggerated AngII‐induced cardiac hypertrophy and fibrosis.^31^ However, it is argued that excessively high levels of Nox4 are likely to result in non‐physiological effects.^32^ Our previous studies using both global Nox4KO and cardiomyocyte‐specific overexpressing mice demonstrated that cardiomyocyte Nox4 is protective against adverse remodelling in response to pressure overload and myocardial infarction.^10^, ^11^, ^12^, ^33^, ^34^ Here, we show that mice overexpressing Nox4 in ECs develop significantly less AngII‐induced myocardial fibrosis without affecting AngII‐induced cardiomyocyte hypertrophy. Moreover, endothelial Nox4 was also found to be protective against interstitial fibrosis in the pressure‐overloaded heart.^35^

Interestingly, we also found a baseline state of modest physiological cardiac hypertrophy in EndoNox4 TG mice. This TG model has a 2.6‐fold increase in endothelial Nox4 protein levels and two‐fold more H_2_O_2_ production than WT, without changes in Nox2 and subunit p22^phox^ expressions.^14^ We found that EndoNox4 TG mice displayed modestly enlarged hearts and an increased IVSD, with a pattern of eccentric hypertrophy similar to physiological cardiac hypertrophy. Consistent with a physiological hypertrophic response, EndoNox4 TG mice had well‐preserved systolic function, by both echocardiography and PV loop analysis, as well as normal diastolic function. Molecular markers of pathological hypertrophy, that is, *Anf*, were unaltered in the EndoNox4 Tg heart. It has long been recognized that cardiac ECs may influence both cardiomyocyte contractile state^36^, ^37^ and cardiac growth, the latter especially during development.^38^, ^39^ The mechanisms underlying Nox4‐dependent physiological cardiomyocyte growth were not investigated in the present study, but there are a number of possibilities. It was reported that H_2_O_2_, the preferential product of Nox4, could release neuregulin 1 from ECs to exert hypertrophic effects on cardiomyocytes.^40^, ^41^ In addition, Nox4 activates eNOS to promote nitric oxide (NO) production,^13^ which could chronically increase coronary blood flow and induce cardiomyocyte growth.^42^ High NO levels from ECs may also promote nearby cardiomyocyte hypertrophy by favouring the degradation of regulator 4 of G‐protein signalling.^43^ Altogether, the presence of physiological cardiac hypertrophy in EndoNox4 TG mice at baseline suggests that ROS‐dependent crosstalk between ECs and cardiomyocytes may influence cardiac growth.

The development of myocardial fibrosis is an important component in the progression of cardiac remodelling to heart failure. ECs play important roles in fibrosis and remodelling through interactions with other cell types, in particular inflammatory cells. This study shows that the up‐regulation of endothelial Nox4 can act to abrogate EC activation and pathological inflammation. The results also indicate complex and distinct roles of Nox2 and Nox4 in modulating fibrosis. Based on the current data, approaches to target Nox‐derived ROS need to be isoform specific.

## Conflict of interest

The authors declare no conflicts of interest.

## Funding

This work was supported by British Heart Foundation grants (RG/20/3/34823 and CH/1999001/11735 to A.M.S. and PG/17/39/33027 to M.Z.). M.W. was funded in part by the China Scholarship Council.

## References

[1] de Boer RA , de Keulenaer G , Bauersachs J , Brutsaert D , Cleland JG , Diez J , Du X‐J , Ford P , Heinzel FR , Lipson KE , McDonagh T , Lopez‐Andres N , Lunde IG , Lyon AR , Pollesello P , Prasad SK , Tocchetti CG , Mayr M , Sluijter JPG , Thum T , Tschöpe C , Zannad F , Zimmermann W‐H , Ruschitzka F , Filippatos G , Lindsey ML , Maack C , Heymans S . Towards better definition, quantification and treatment of fibrosis in heart failure. A scientific roadmap by the Committee of Translational Research of the Heart Failure Association (HFA) of the European Society of Cardiology. Eur J Heart Fail 2019: 272–285. 30714667 10.1002/ejhf.1406 PMC6607480

[2] Gyöngyösi M , Winkler J , Ramos I , Do Q‐T , Firat H , McDonald K , González A , Thum T , Díez J , Jaisser F , Pizard A , Zannad F . Myocardial fibrosis: biomedical research from bench to bedside. Eur J Heart Fail 2017; 19: 177–191. 28157267 10.1002/ejhf.696 PMC5299507

[3] Cook‐Mills JMMM , Abdala‐Valencia H . Vascular cell adhesion molecule‐1 expression and signaling during disease: regulation by reactive oxygen species and antioxidants. Antioxid Redox Signal 2011; 15: 1607–1638. 21050132 10.1089/ars.2010.3522 PMC3151426

[4] Nguyen Dinh Cat A , Montezano AC , Burger D , Touyz RM . Angiotensin II, NADPH oxidase, and redox signaling in the vasculature. Antioxid Redox Signal 2013; 19: 1110–1120. 22530599 10.1089/ars.2012.4641 PMC3771549

[5] Bendall JK , Cave AC , Heymes C , Gall N , Shah AM . Pivotal role of a gp91(phox)‐containing NADPH oxidase in angiotensin II‐induced cardiac hypertrophy in mice. Circulation 2002; 105: 293–296. 11804982 10.1161/hc0302.103712

[6] Johar S , Cave AC , Narayanapanicker A , Grieve DJ , Shah AM . Aldosterone mediates angiotensin II‐induced interstitial cardiac fibrosis via a Nox2‐containing NADPH oxidase. FASEB J 2006; 20: 1546–1548. 16720735 10.1096/fj.05-4642fje

[7] Satoh M , Ogita H , Takeshita K , Mukai Y , Kwiatkowski DJ , Liao JK . Requirement of Rac1 in the development of cardiac hypertrophy. Proc Natl Acad Sci 2006; 103: 7432–7437. 16651530 10.1073/pnas.0510444103 PMC1455410

[8] Murdoch CE , Chaubey S , Zeng L , Yu B , Ivetic A , Walker SJ , Vanhoutte D , Heymans S , Grieve DJ , Cave AC , Brewer AC , Zhang M , Shah AM . Endothelial NADPH oxidase‐2 promotes interstitial cardiac fibrosis and diastolic dysfunction through proinflammatory effects and endothelial‐mesenchymal transition. J Am Coll Cardiol 2014; 63: 2734–2741. 24681145 10.1016/j.jacc.2014.02.572

[9] Zhang M , Perino A , Ghigo A , Hirsch E , Shah AM . NADPH oxidases in heart failure: poachers or gamekeepers? Antioxid Redox Signal 2013; 18: 1024–1041. 22747566 10.1089/ars.2012.4550 PMC3567780

[10] Zhang M , Brewer AC , Schröder K , Santos CXC , Grieve DJ , Wang M , Anilkumar N , Yu B , Dong X , Walker SJ , Brandes RP , Shah AM . NADPH oxidase‐4 mediates protection against chronic load‐induced stress in mouse hearts by enhancing angiogenesis. Proc Natl Acad Sci 2010; 107: 18121–18126. 20921387 10.1073/pnas.1009700107 PMC2964252

[11] Smyrnias I , Zhang X , Zhang M , Murray TVA , Brandes RP , Schröder K , Brewer AC , Shah AM . Nicotinamide adenine dinucleotide phosphate oxidase‐4‐dependent upregulation of nuclear factor erythroid‐derived 2‐like 2 protects the heart during chronic pressure overload. Hypertension 2015; 65: 547–553. 25534702 10.1161/HYPERTENSIONAHA.114.04208

[12] Santos CX , Hafstad AD , Beretta M , Zhang M , Molenaar C , Kopec J , Fotinou D , Murray TV , Cobb AM , Martin D , Zeh Silva M , Anilkumar N , Schröder K , Shanahan CM , Brewer AC , Brandes RP , Blanc E , Parsons M , Belousov V , Cammack R , Hider RC , Steiner RA , Shah AM . Targeted redox inhibition of protein phosphatase 1 by Nox4 regulates eIF2‐mediated stress signaling. EMBO J 2016; 35: 319–334. 26742780 10.15252/embj.201592394 PMC4741303

[13] Schröder K , Zhang M , Benkhoff S , Mieth A , Pliquett R , Kosowski J , Kruse C , Luedike P , Michaelis UR , Weissmann N , Dimmeler S , Shah AM , Brandes RP . Nox4 is a protective reactive oxygen species generating vascular NADPH oxidase. Circ Res 2012; 110: 1217–1225. 22456182 10.1161/CIRCRESAHA.112.267054

[14] Ray R , Murdoch CE , Wang M , Santos CX , Zhang M , Alom‐Ruiz S , Anilkumar N , Ouattara A , Cave AC , Walker SJ , Grieve DJ , Charles RL , Eaton P , Brewer AC , Shah AM . Endothelial Nox4 NADPH oxidase enhances vasodilatation and reduces blood pressure in vivo. Arterioscler Thromb Vasc Biol 2011; 31: 1368–1376. 21415386 10.1161/ATVBAHA.110.219238

[15] Zhang M , Prosser BL , Bamboye MA , Gondim ANS , Santos CX , Martin D , Ghigo A , Perino A , Brewer AC , Ward CW , Hirsch E , Lederer WJ , Shah AM . Contractile function during angiotensin‐II activation: increased Nox2 activity modulates cardiac calcium handling via phospholamban phosphorylation. J Am Coll Cardiol 2015; 66: 261–272. 26184620 10.1016/j.jacc.2015.05.020 PMC4509515

[16] Murray TVA , Smyrnias I , Schnelle M , Mistry RK , Zhang M , Beretta M , Martin D , Anilkumar N , de Silva SM , Shah AM , Brewer AC . Redox regulation of cardiomyocyte cell cycling via an ERK1/2 and c‐Myc‐dependent activation of cyclin D2 transcription. J Mol Cell Cardiol 2015; 79: 54–68. 25450615 10.1016/j.yjmcc.2014.10.017 PMC4312357

[17] Zakkar M , Chaudhury H , Sandvik G , Enesa K , Luong LA , Cuhlmann S , Mason JC , Krams R , Clark AR , Haskard DO , Evans PC . Increased endothelial mitogen‐activated protein kinase phosphatase‐1 expression suppresses proinflammatory activation at sites that are resistant to atherosclerosis. Circ Res 2008; 103: 726–732. 18723442 10.1161/CIRCRESAHA.108.183913

[18] Schnelle M , Sawyer I , Anilkumar N , Mohamed BA , Richards DA , Toischer K , Zhang M , Catibog N , Sawyer G , Mongue‐Din H , Schröder K , Hasenfuss G , Shah AM . NADPH oxidase‐4 promotes eccentric cardiac hypertrophy in response to volume overload. Cardiovasc Res 2019: cvz331. 10.1093/cvr/cvz331 PMC7797217 31821410

[19] Godo S , Shimokawa H . Endothelial functions. Arterioscler Thromb Vasc Biol 2017; 37: e108–e114. 28835487 10.1161/ATVBAHA.117.309813

[20] Pober JS , Sessa WC . Evolving functions of endothelial cells in inflammation. Nat Rev Immunol 2007; 7: 803–815. 17893694 10.1038/nri2171

[21] Matheny HE , Deem TL , Cook‐Mills JM . Lymphocyte migration through monolayers of endothelial cell lines involves VCAM‐1 signaling via endothelial cell NADPH oxidase. J Immunol 2000; 164: 6550–6559. 10843714 10.4049/jimmunol.164.12.6550

[22] Murdoch C , Alom‐Ruiz S , Wang M , Zhang M , Walker S , Yu B , Brewer A , Shah A . Role of endothelial Nox2 NADPH oxidase in angiotensin II‐induced hypertension and vasomotor dysfunction. Basic Res Cardiol 2011: 1–12. 21528437 10.1007/s00395-011-0179-7 PMC3105229

[23] Mylroie H , Dumont O , Bauer A , Thornton CC , Mackey J , Calay D , Hamdulay SS , Choo JR , Boyle JJ , Samarel AM , Randi AM , Evans PC , Mason JC . PKCε‐CREB‐Nrf2 signalling induces HO‐1 in the vascular endothelium and enhances resistance to inflammation and apoptosis. Cardiovasc Res 2015; 106: 509–519. 25883219 10.1093/cvr/cvv131 PMC4431664

[24] Al‐Rashed F , Calay D , Lang M , Thornton CC , Bauer A , Kiprianos A , Haskard DO , Seneviratne A , Boyle JJ , Schönthal AH , Wheeler‐Jones CP , Mason JC . Celecoxib exerts protective effects in the vascular endothelium via COX‐2‐independent activation of AMPK‐CREB‐Nrf2 signalling. Sci Rep 2018; 8: 6271. 29674687 10.1038/s41598-018-24548-z PMC5908847

[25] Hancock M , Hafstad AD , Nabeebaccus AA , Catibog N , Logan A , Smyrnias I , Hansen SS , Lanner J , Schroder K , Murphy MP , Shah AM , Zhang M . Myocardial NADPH oxidase‐4 regulates the physiological response to acute exercise. Elife 2018; 7. 10.7554/eLife.41044 PMC6307857 30589411

[26] Touyz RM , Mercure C , He Y , Javeshghani D , Yao G , Callera GE , Yogi A , Lochard N , Reudelhuber TL . Angiotensin II‐dependent chronic hypertension and cardiac hypertrophy are unaffected by gp91phox‐containing NADPH oxidase. Hypertension 2005; 45: 530–537. 15753233 10.1161/01.HYP.0000158845.49943.5e

[27] Emmerson A , Trevelin SC , Mongue‐Din H , Becker PD , Ortiz C , Smyth LA , Peng Q , Elgueta R , Sawyer G , Ivetic A , Lechler RI , Lombardi G , Shah AM . Nox2 in regulatory T cells promotes angiotensin II‐induced cardiovascular remodeling. J Clin Invest 2018; 128: 3088–3101. 29688896 10.1172/JCI97490 PMC6025997

[28] Siddesha JM , Valente AJ , Sakamuri SSVP , Yoshida T , Gardner JD , Somanna N , Takahashi C , Noda M , Chandrasekar B . Angiotensin II stimulates cardiac fibroblast migration via the differential regulation of matrixins and RECK. J Mol Cell Cardiol 2013; 65: 9–18. 24095877 10.1016/j.yjmcc.2013.09.015 PMC3896127

[29] Mummidi S , Das NA , Carpenter AJ , Kandikattu H , Krenz M , Siebenlist U , Valente AJ , Chandrasekar B . Metformin inhibits aldosterone‐induced cardiac fibroblast activation, migration and proliferation in vitro, and reverses aldosterone + salt‐induced cardiac fibrosis in vivo. J Mol Cell Cardiol 2016; 98: 95–102. 27423273 10.1016/j.yjmcc.2016.07.006

[30] Cucoranu I , Clempus R , Dikalova A , Phelan PJ , Ariyan S , Dikalov S , Sorescu D . NAD(P)H oxidase 4 mediates transforming growth factor‐β1‐induced differentiation of cardiac fibroblasts into myofibroblasts. Circ Res 2005; 97: 900–907. 16179589 10.1161/01.RES.0000187457.24338.3D

[31] Zhao QD , Viswanadhapalli S , Williams P , Shi Q , Tan C , Yi X , Bhandari B , Abboud HE . NADPH oxidase 4 induces cardiac fibrosis and hypertrophy through activating Akt/mTOR and NFκB signaling pathways. Circulation 2015; 131: 643–655. 25589557 10.1161/CIRCULATIONAHA.114.011079 PMC4568756

[32] Shah AM . Parsing the role of NADPH oxidase enzymes and reactive oxygen species in heart failure. Circulation 2015; 131: 602–604. 25589558 10.1161/CIRCULATIONAHA.115.014906

[33] Mongue‐Din H , Patel AS , Looi YH , Grieve DJ , Anilkumar N , Sirker A , Dong X , Brewer AC , Zhang M , Smith A , Shah AM . NADPH oxidase‐4 driven cardiac macrophage polarization protects against myocardial infarction–induced remodeling. JACC: Basic to Translational Science 2017; 2: 688–698. 29445778 10.1016/j.jacbts.2017.06.006 PMC5803556

[34] Nabeebaccus AA , Zoccarato A , Hafstad AD , Santos CXC , Aasum E , Brewer AC , Zhang M , Beretta M , Yin X , West JA , Schröder K , Griffin JL , Eykyn TR , Abel ED , Mayr M , Shah AM . Nox4 reprograms cardiac substrate metabolism via protein O‐GlcNAcylation to enhance stress adaptation. JCI Insight 2017; 2. 10.1172/jci.insight.96184 PMC5752273 29263294

[35] Zhang M , Mongue‐Din H , Martin D , Catibog N , Smyrnias I , Zhang X , Yu B , Wang M , Brandes RP , Schröder K , Shah AM . Both cardiomyocyte and endothelial cell Nox4 mediate protection against hemodynamic overload‐induced remodelling. Cardiovasc Res 2018; 114: 401–408. 29040462 10.1093/cvr/cvx204 PMC6018755

[36] Brutsaert DL , Meulemans AL , Sipido KR , Sys SU . Effects of damaging the endocardial surface on the mechanical performance of isolated cardiac muscle. Circ Res 1988; 62: 358–366. 3338120 10.1161/01.res.62.2.358

[37] Paulus WJ , Vantrimpont PJ , Shah AM . Paracrine coronary endothelial control of left ventricular function in humans. Circulation 1995; 92: 2119–2126. 7554191 10.1161/01.cir.92.8.2119

[38] Zhang M , Shah AM . ROS signalling between endothelial cells and cardiac cells. Cardiovasc Res 2014; 102: 249–257. 24591150 10.1093/cvr/cvu050

[39] Segers VFM , Brutsaert DL , De Keulenaer GW . Cardiac remodeling: endothelial cells have more to say than just NO. Front Physiol 2018; 9. 10.3389/fphys.2018.00382 PMC5904256 29695980

[40] Lemmens K , Segers VFM , Demolder M , De Keulenaer GW . Role of neuregulin‐1/ErbB2 signaling in endothelium‐cardiomyocyte cross‐talk. J Biol Chem 2006; 281: 19469–19477. 16698793 10.1074/jbc.M600399200

[41] Kuramochi Y , Cote GM , Guo X , Lebrasseur NK , Cui L , Liao R , Sawyer DB . Cardiac endothelial cells regulate reactive oxygen species‐induced cardiomyocyte apoptosis through neuregulin‐1β/erbB4 signaling. J Biol Chem 2004; 279: 51141–51147. 15385548 10.1074/jbc.M408662200

[42] Tirziu D , Chorianopoulos E , Moodie KL , Palac RT , Zhuang ZW , Tjwa M , Roncal C , Eriksson U , Fu Q , Elfenbein A , Hall AE , Carmeliet P , Moons L , Simons M . Myocardial hypertrophy in the absence of external stimuli is induced by angiogenesis in mice. J Clin Invest 2007; 117: 3188–3197. 17975666 10.1172/JCI32024 PMC2045601

[43] Jaba IM , Zhuang ZW , Li N , Jiang Y , Martin KA , Sinusas AJ , Papademetris X , Simons M , Sessa WC , Young LH , Tirziu D . NO triggers RGS4 degradation to coordinate angiogenesis and cardiomyocyte growth. J Clin Invest 2013; 123: 1718–1731. 23454748 10.1172/JCI65112 PMC3613910

